# The Therapeutic Efficacy of Abatacept for Rheumatoid Arthritis-Associated Interstitial Lung Disease: Insights from a 12-Month Trial Using Semi-Quantitative Chest High-Resolution Computed Tomography Imaging

**DOI:** 10.3390/jcm13195871

**Published:** 2024-10-01

**Authors:** Takeshi Shoda, Takuya Kotani, Mitsuhiro Koyama, Ayaka Yoshikawa, Yumiko Wada, Hidehiko Makino, Keigo Osuga, Tohru Takeuchi

**Affiliations:** 1 Department of Internal Medicine (IV), Division of Rheumatology, Osaka Medical and Pharmaceutical University, 2-7 Daigaku-machi, Takatsuki City 569-8686, Osaka, Japan; takeshi.shoda@ompu.ac.jp (T.S.); ayaka.yoshikawa@ompu.ac.jp (A.Y.); yumiko.wada@ompu.ac.jp (Y.W.); hidehiko.makino@ompu.ac.jp (H.M.); tooru.takeuchi@ompu.ac.jp (T.T.); 2 Department of Diagnostic Radiology, Osaka Medical and Pharmaceutical University, 2-7 Daigaku-machi, Takatsuki City 569-8686, Osaka, Japan; mitsuhiro.koyama@ompu.ac.jp (M.K.); osuga@ompu.ac.jp (K.O.)

**Keywords:** abatacept, chest HRCT scoring, interstitial lung disease, rheumatoid arthritis

## Abstract

**Background:** Rheumatoid arthritis-associated interstitial lung disease (RA-ILD) is a major complication of rheumatoid arthritis (RA), but effective treatment remains an unmet need in its management. Our aim was to evaluate the therapeutic efficacy of abatacept for RA-ILD. **Methods:** This observational retrospective study included patients with RA-ILD treated with abatacept between 2012 and 2021. Indices of RA disease activity and interstitial lung disease (Disease Activity Score in 28 joints using C-reactive Protein [DAS28-CRP], Simplified Disease Activity Index [SDAI], Clinical Disease Activity Index [CDAI], serum Krebs von den Lungen-6 levels, % forced vital capacity [%FVC], and semi-quantified chest high-resolution computed tomography scores) were evaluated before and 1 year after the start of abatacept administration. **Results:** Overall, 38 patients were included. DAS28-CRP, SDAI, and CDAI were significantly improved (all with *p* < 0.0001). Total ground-glass opacity scores were decreased in both patients with usual interstitial pneumonia (UIP)-like patterns and with non-UIP-like patterns (*p* = 0.008 and <0.002, respectively). Total fibrosis scores were also decreased in the UIP-like pattern group (*p* < 0.042). The %FVC remained stable. **Conclusions:** Abatacept significantly improves RA disease activity and reduces pulmonary inflammation in patients with RA-ILD.

## 1. Introduction

Rheumatoid arthritis (RA) is a form of systemic immune-related arthritis often associated with lung complications such as interstitial lung disease (ILD) and airway diseases [1]. ILD is a major complication in patients with RA, with a high mortality rate [2,3]. ILD is prevalent in 28–67% of patients with RA, with a worse prognosis than in those without ILD [4]. RA with ILD is difficult to treat because the use of disease-modifying antirheumatic drugs (DMARDs) is limited in these patients; thus, effective treatment is an unmet need in RA management [5]. The clinical course of RA-ILD is heterogeneous, with varying ILD patterns. ILD patterns in RA can be mainly classified into usual interstitial pneumonia (UIP)-like and nonspecific interstitial pneumonia (NSIP)-like patterns [6]. The UIP-like pattern is more prevalent in RA compared with other connective tissue diseases [7], and RA-associated UIP-like ILD has a worse prognosis than NSIP-like patterns in RA. The pathogenesis of RA and related ILD remains unclear; however, anti-citrullinated protein antibodies (ACPAs) are related not only to arthritis but also to ILD. Citrullinated proteins have been observed in both articular and lung lesions in RA [8]. Arthritis may also be closely associated with ILD.

The treatment of RA-ILD is controversial because the choice of therapeutic agents remains challenging. Abatacept (ABT) is a unique biological DMARD (bDMARD) that inhibits T lymphocyte costimulation and is therapeutically effective against moderate-to-severe RA, especially ACPA-positive RA [9]. Recent reports have shown favorable responses to ABT in RA-ILD [10]. British and American guidelines also conditionally recommend ABT for the treatment of RA-ILD [11]. However, patients with RA-ILD have varying characteristics, including age, lung function, ILD patterns, and treatment response. Evidence of the effectiveness of ABT in these patients is limited.

Thus, this study aimed to evaluate the therapeutic efficacy of ABT in patients with RA-ILD. To achieve this goal, RA disease activity indicators and ILD-related indicators, particularly semi-quantified chest high-resolution computed tomography (HRCT) imaging findings, were compared before and after ABT administration.

## 2. Materials and Methods

### 2.1. Study Design and Patients

This observational retrospective study was conducted in accordance with the Declaration of Helsinki and its amendments and was approved by the Medicine Ethics Committee of Osaka Medical and Pharmaceutical University (approval no. 1529, 1598). Informed consent was obtained from all patients.

Consecutive patients diagnosed with RA-ILD and treated with ABT between November 2012 and March 2021 in Osaka Medical and Pharmaceutical University Hospital were evaluated. The inclusion criterion was the availability of data in the form of RA disease activity indices and chest HRCT findings from both before and after 1 year of treatment. RA diagnosis was based on the 2010 ACR/European League Against Rheumatism classification criteria [12]. ILD diagnosis was made according to the American Thoracic Society/European Respiratory Society 2013 criteria [13] and these were applied by radiologists based on the chest HRCT findings.

### 2.2. Assessment and Outcomes

Baseline demographic data, including age, sex, duration of RA, smoking history, rheumatoid factor (RF) positivity/titer (reference range: <15 IU/mL), ACPA positivity/titer (reference range: <4.5 U/mL), disease activity indicators (Disease Activity Score in 28 joints using C-reactive Protein [DAS28-CRP], Simplified Disease Activity Index [SDAI], and Clinical Disease Activity Index [CDAI]), serum levels of Krebs von den Lungen-6 (KL-6) (reference range: 105–401 U/mL), the % forced vital capacity (%FVC), chest HRCT scores, and treatment details were collected. Data on RA disease activity indicators, serum KL-6 levels, the %FVC, and chest HRCT scores 1 year after ABT initiation were also obtained.

### 2.3. Evaluation of Chest HRCT Patterns and Scores

Chest HRCT was performed using a 64-detector row CT multiscanner at Osaka Medical and Pharmaceutical University Hospital (Aquilon; Toshiba Medical Systems Corporation, Tokyo, Japan). The slice thickness was 1.0–1.5 mm every 10 mm, and the entire lung was covered. All patients underwent chest HRCT before and 1 year after treatment initiation. CT images were independently reviewed by three observers blinded to the clinical patient information. The three observers comprised two radiologists specializing in pulmonary imaging (M.K. and K.O.) and one pulmonologist (T.S.). Inter-observer disagreements were resolved by consensus. The chest HRCT findings were classified into UIP-like and non-UIP-like patterns based on previous reports [13,14]. Ground-glass opacity (GGO) and fibrosis were scored to assess the HRCT findings, as previously described [15]. Each lobe was scored by the same observer, and the average value was used. The scores were then summed to obtain the total CT score. Briefly, three limited CT levels were preselected: mid-aortic arch, left tracheal bifurcation, and 1 cm above the diaphragm. Each lobe (right upper, middle, and lower; and left upper and lower lobes) of the lungs was semi-quantitatively scored at the three sites according to GGO, septal thickening, and honeycombing. GGO scores were generated on a scale of 0–5 according to the extent of GGO involvement in the lobe, as follows: no involvement, 0; ≤ 5%, 1; 5% to <25%, 2; 25–49%, 3; 50–75%, 4; and >75%, 5. Fibrosis scores were generated according to the extent of the incidence of honeycombing in the lobe, as follows: no incidence, 0; interlobular septal thickening without discrete honeycombing, 1; <25%, 2; 25–49%, 3; 50–75%, 4; and >75%, 5.

### 2.4. Statistical Analysis

The data are presented as the median (interquartile range). Fisher’s exact test was used where appropriate, and the Mann–Whitney U-test was used to compare the median values. Paired analyses of the disease activity indicators of RA and the quantitative indices related to interstitial lung disease before and 1 year after ABT initiation were assessed using Wilcoxon’s signed-rank test. Univariate and multivariate logistic regression analyses were conducted to assess the effects of glucocorticoids (GCs), tacrolimus (TAC), methotrexate (MTX), RF positivity, and smoking history on GGO reduction in patients with RA-ILD. All statistical analyses were performed using JMP pro version 16.2 (SAS Institute Inc., Cary, NC, USA). Statistical significance was set at *p* < 0.05.

## 3. Results

### 3.1. Baseline Characteristics and Treatment Details

Overall, 38 consecutive patients were included in this study. The baseline characteristics and treatment details are shown in Table 1. The median age was 73 (range, 67–78.3) years, and 63% were female. In total, 18 (47%) patients had a smoking history. The median duration of RA was 41.2 (range, 9.6–134.2) weeks. In total, 84% (*n* = 32) had RF positivity, and the median titer was 191 (range, 59.4–598) IU/mL, while 87% (*n*= 33) had ACPA positivity, and the median titer was 336 (range, 146.5–500) U/mL. The median DAS28-CRP, SDAI, and CDAI scores were 3.7 (range, 3–4.2), 14.8 (range, 11.3–19.7), and 13.8 (range, 9.3–18.7), respectively. For the chest HRCT pattern, 16 patients (42%) had a UIP-like pattern, and 22 (58%) patients had a non-UIP-like pattern. In the non-UIP-like group, 6 (16%) patients had NSIP, whereas 16 (42%) patients had other patterns. Regarding ILD indicators, the median serum KL-6 levels, %FVC, total GGO score, and total fibrosis score were 428 U/mL (range, 274–643), 95.1% (range, 81–106.7), 5.2 (range, 3–7.8), and 3.3 (range, 2–5.3), respectively. The concomitant medications used were MTX in 26% (median dose: 7.5 [range, 4–9.3] mg/week); TAC in 58%; GCs in 34% (median dose: 5 [range, 5–10] mg/day); salazosulfapyridine in 39%; bucillamine in 7.9%; iguratimod in 7.9%; and azathioprine in 5.3% of the patients.

### 3.2. Comparison of Baseline Clinical Characteristics and Treatment Regimens between UIP-like Pattern and Non-UIP-like Pattern Groups

The comparison of baseline clinical characteristics and treatment regimens between the two groups is shown in Table 2. RF positivity was significantly higher in the UIP-like pattern group than in the non-UIP-like pattern group (*p* = 0.030). The median total fibrosis score was significantly higher in the UIP-like pattern group than in the non-UIP-like pattern group (*p* = 0.0003).

### 3.3. Therapeutic Effects of Abatacept in Patients with RA-ILD

**Figure 1:**
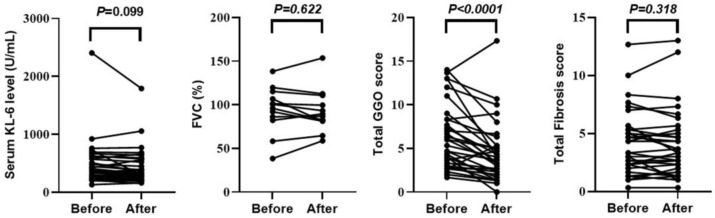
Changes in ILD indicators before and 1 year after the start of ABT administration. Paired analysis is performed using Wilcoxon’s signed-rank test. Statistical significance is set at *p* < 0.05. Before, before ABT administration; After, 1 year after the start of ABT administration; KL-6, Krebs von den Lungen-6; FVC, forced vital capacity; GGO, ground-glass opacity.

**Figure 2:**
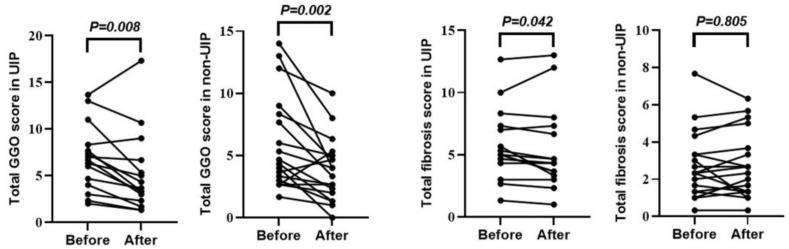
Changes in chest HRCT scores before and 1 year after the start of ABT administration categorized by UIP-like and non-UIP-like patterns. Paired analysis is performed using Wilcoxon’s signed-rank test. Statistical significance is set at *p* < 0.05. ABT, abatacept; UIP, usual interstitial pneumonia-like pattern; non-UIP, non-UIP-like pattern; Before, before ABT administration; After, 1 year after the start of ABT administration; KL-6, Krebs von den Lungen-6; FVC, forced vital capacity; GGO, ground-glass opacity.

**Figure 3:**
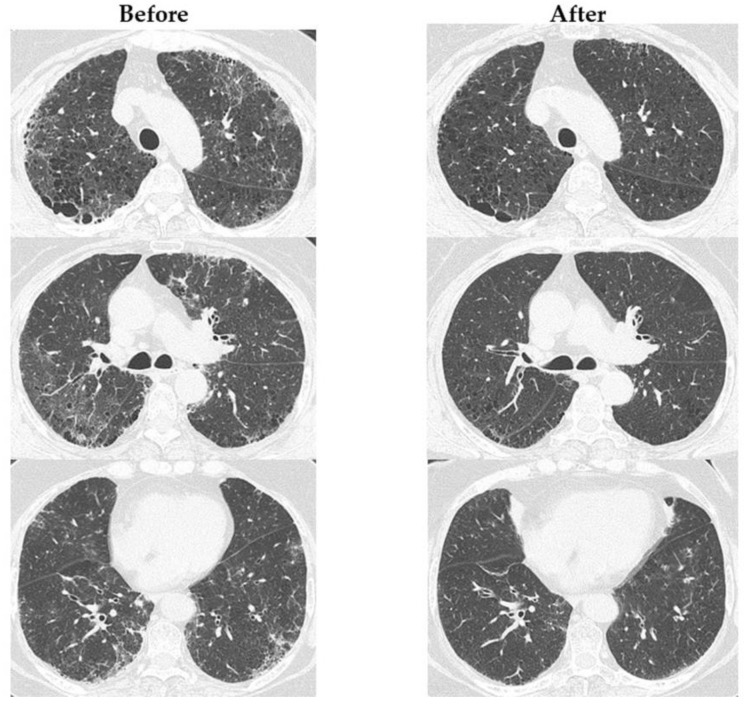
An example case of changes in chest HRCT findings before and 1 year after the start of ABT administration. The images were evaluated at three CT levels: the aortic arch, the left tracheal bifurcation, and 1 cm above the diaphragm. The extent of GGO, septal thickening, and honeycombing in each lung lobe (right upper lobe, middle lobe, lower lobe, left upper lobe, and left lower lobe) was semi-quantitatively assessed in the three images. The total GGO score improved from 11 to 5.33 points, and the total fibrosis score improved from 5.67 to 3.33 points. Before: before ABT administration; After: 1 year after the start of ABT administration; GGO: ground-glass opacity.

Figure S1 shows the change in RA disease activity before and 1 year after the start of ABT administration. The following indicators were significantly improved after 1 year’s administration of ABT: DAS28CRP, 2.0 (range, 1.6–2.6); SDAI, 4.2 (range, 1.7–8.3); and CDAI, 3.9 (range, 1–7.8) (all *p* < 0.0001). Figure 1 shows the changes in ILD indicators before and 1 year after the start of ABT administration. The total GGO score (3.7 [range, 2.2–5.3]) was significantly decreased (*p* < 0.0001) after 1 year of ABT administration. Figure 2 shows the changes in chest HRCT scores, categorized by UIP-like and non-UIP-like patterns, before and 1 year after the start of ABT administration. The following values were significantly lower after 1 year of ABT administration: the total GGO score in the UIP-like pattern group (3.7 [range, 2.3–6.7], *p* = 0.008); the total GGO score in the non-UIP-like pattern group (3.6 [range, 1.8–5.1], *p* < 0.002); and the total fibrosis score in the UIP-like pattern group (4.3 [range, 3.3–7.3], *p* < 0.042). Figure S2 shows the changes in chest HRCT scores, categorized by MTX use, non-use, smoking, and non-smoking, before and 1 year after the start of ABT administration. There is no difference in the abatacept response between these categorized groups. Figure 3 shows an example case of changes in chest HRCT findings before and 1 year after the start of ABT administration.

### 3.4. Logistic Regression Analyses of GGO Reduction in Chest HRCT Findings

Except for ABT, the use of GCs, TAC, and MTX, RF positivity, and smoking history may contribute to the reduction in GGO on the chest HRCT images. However, univariate and multivariate logistic regression analyses showed that the use of GCs, TAC, and MTX, RF positivity, and smoking history did not significantly contribute to the reduction in GGO in the chest HRCT findings (Table 3).

## 4. Discussion

This study found that ABT significantly improved RA disease activity indices 1 year after its initiation. Further, ILD indicators, including serum KL-6 levels and the total GGO score, were also decreased, whereas the %FVC and total fibrosis score showed no significant changes. For chest HRCT scores, the total GGO score was significantly decreased in both the UIP-like pattern and non-UIP-like pattern groups. The total fibrosis score was also significantly decreased in the UIP-like pattern group. Logistic regression analyses indicated that ABT, but not GCs, TAC, MTX, RF positivity, or smoking history, significantly contributed to GGO reduction on chest HRCT. These findings provide a deeper understanding of the effectiveness of ABT in the treatment of RA-ILD.

The effect of ABT on pulmonary lesions in patients with RA-ILD has been extensively studied [10,16,17,18,19,20,21,22,23,24,25,26,27]. Cassone et al. conducted a systematic review and reported that ABT led to improvement in 16.6% of cases, stabilization in 74.9% of cases, and deterioration in 8.5% of cases [10]. Further studies evaluated the effects of ABT on pulmonary function in patients with RA-ILD, with the population size ranging from 39 to 163 patients, and the median follow-up period ranging from 12 to 24 months. The results demonstrated that the %FVC improved in 8.3–12%, stabilized in 77.8–87.7%, and worsened in 12–13.9% of the cases. Meanwhile, the %DLco improved in 14–30.5%, stabilized in 58.3–75%, and deteriorated in 9–11.1% of the cases [10,21,24,25]. Chest HRCT studies involving 44–128 patients during an observation period of approximately 12–27.3 months post-ABT initiation indicated improvement in 8.8%–18.8%, stabilization in 57.8–72.7%, and deterioration in 11.3–23.4% of the cases [10,21,24-27]. A previous comparative analysis between UIP- and non-UIP-like patterns in patients with RA-ILD treated with ABT showed no significant differences in pulmonary function or chest HRCT findings [23]. Notably, chest HRCT images were evaluated for improvement, stabilization, and deterioration through radiologist consensus; however, quantitative measures were not employed in these evaluations. Previous reports have documented the stabilization of pulmonary function, and our study further confirms the stability of the %FVC. With respect to chest HRCT findings, we semi-quantitatively evaluated both inflammatory (GGO) and fibrotic changes and found a significant reduction in the total GGO score. Although improvements in chest HRCT findings have been reported, our study elucidated specific improvements using a semi-quantitative approach. Additionally, the significant reduction in the total fibrosis score in the UIP-like pattern group presents new insights that have not been highlighted previously.

However, the therapeutic mechanisms of ABT in RA-ILD remain unclear. RA is an autoimmune disease caused by citrullinated proteins in synovial joints, and a similar mechanism is thought to occur in lung lesions [28]. ABT may inhibit T-lymphocyte-mediated inflammation in the lungs and joints, resulting in reduced GGO levels that reflect lung inflammation. Both GGO and fibrosis scores are decreased in RA-UIP, as in the present study. Although UIP is predominantly fibrotic, RA-UIP is slightly different from idiopathic pulmonary fibrosis in pathological specimens and may present with inflammatory lesions [29]. Septal thickening was included as part of the fibrosis score in the chest HRCT scoring system [15] used in this study. However, septal thickening observed in chest images can also result from the infiltration of inflammatory cells into pulmonary lesions [30]. Therefore, the decreased fibrosis scores observed in the RA UIP-like pattern group may also reflect the suppression of pulmonary inflammation. Further detailed investigations are needed to determine whether ABT suppresses pulmonary fibrosis in RA-UIP.

ABT significantly reduced the DAS28-ESR and CDAI scores in previous studies involving 16–263 patients with RA-ILD observed over a period of 12–27.3 months [20,22,24,25,26,27]. In our study, the DAS28-CRP, SDAI, and CDAI scores were significantly decreased 1 year after the start of ABT administration, supporting these previous findings. ABT appears to be effective in suppressing RA disease activity in patients with RA-ILD. RA-ILD is treated with immunosuppressive agents such as GCs and TAC [31]. Although these agents were used to treat RA in this study, no additional or increased doses were administered to treat ILD. The multivariate analysis also showed that these drugs did not contribute to the therapeutic effects of ABT in RA-ILD. In particular, GCs and TAC had minimal therapeutic effects on ILD.

Recent reports suggest that MTX does not influence the progression of RA-ILD [32,33]. However, in the decision-making strategy for RA-ILD proposed by experts [4], the use of MTX is discouraged in cases of clinically evident ILD due to the risk of MTX-induced pulmonary toxicity. Furthermore, even in subclinical ILD, the use of MTX should be avoided if there are risk factors for MTX-induced pulmonary toxicity or acute ILD exacerbation (e.g., undernutrition, chronic renal failure, reduced pulmonary function, or radiological honeycombing). Given these potential negative impacts of MTX on RA-ILD, it is often preferable to select csDMARDs other than MTX and/or ABT for the treatment of RA-ILD.

Although this study provides valuable insights into the therapeutic effects of ABT in patients with RA-ILD, it also has some limitations. First, the study design was observational and retrospective, and this may have introduced selection bias and limited the generalizability of the findings. Additionally, the sample size was relatively small, which may have affected the statistical power and precision of the results. Furthermore, this study was conducted at a single center, potentially limiting the external validity of the findings to other settings or populations. Moreover, the lack of a control group receiving placebo or alternative treatments makes it challenging to establish causality or to compare the efficacy of ABT with that of other therapeutic modalities. Finally, the follow-up period was limited to 1 year, and the long-term outcomes and safety profiles of ABT were not assessed. Therefore, the results should be interpreted cautiously. Nonetheless, the strength of this study lies in the detailed semi-quantitative evaluation of chest HRCT images, which demonstrate the significant effect of ABT treatment on inflammation in the pulmonary lesions of patients with RA-ILD. Further large-scale prospective studies are warranted to address the limitations and confirm the therapeutic benefits of ABT in patients with RA-ILD.

## 5. Conclusions

ABT significantly inhibits RA disease activity and reduces pulmonary inflammation in patients with RA-ILD. Additionally, the reduction in fibrosis scores observed in the UIP-like pattern group suggests that ABT may exert beneficial effects on lobular septal thickening, which is induced by the infiltration of inflammatory cells. The therapeutic effects of ABT are likely attributed to its inhibition of T-lymphocyte costimulation, which plays a role in suppressing inflammation in both the joints and lungs, thus providing a comprehensive treatment benefit for patients with RA-ILD.

## Figures and Tables

**Table 1:** Patient and treatment characteristics (*n* = 38).

Characteristic	Values
Age, years	73 (67–78.3)
Female sex, *n* (%)	24 (63)
Smoking history, *n* (%)	18 (47)
Disease duration of RA, months	41.2 (9.6–134.2)
RF positivity, *n* (%)	32 (84)
RF, IU/mL	191 (59.4–598)
ACPA positivity, *n* (%)	33 (87)
ACPA, U/mL	336 (146.5–500)
RA disease activity indicator:	
DAS28-CRP (*n* = 36)	3.7 (3.0–4.2)
SDAI (*n* = 36)	14.8 (11.3–19.7)
CDAI (*n* = 36)	13.8 (9.3–18.7)
Chest HRCT pattern:	
UIP-like, *n* (%)	16 (42)
Non-UIP-like, *n* (%)	22 (58)
NSIP-like, *n* (%)	6 (16)
Others, *n* (%)	16 (42)
ILD indicators:	
KL-6 (*n* = 32), U/mL	428 (274–643)
FVC (*n* = 23), %	95.1 (81–106.7)
Total GGO score	5.2 (3–7.8)
Total fibrosis score	3.3 (2–5.3)
Treatments:	
MTX, *n* (%)	10 (26)
MTX dose, mg/week	7.5 (4–9.3)
TAC, *n* (%)	22 (58)
GCs, *n* (%)	13 (34)
GC dose, mg/day	5 (5–10)
Others, *n* (%)	SASP, 15 (39); Buc, 3 (7.9); IGU, 3 (7.9); AZA, 2 (5.3)

Continuous variables are shown as the median (interquartile range). RA, rheumatoid arthritis; ILD, interstitial lung disease; RF, rheumatoid factor; ACPA, anti-cyclic citrullinated peptides antibody; CRP, C-reactive protein; DAS28-CRP, Disease Activity Score in 28 Joints using C-reactive Protein; SDAI, Simplified Disease Activity Index; CDAI, Clinical Disease Activity Index; KL-6, Krebs von den Lungen-6; MTX, methotrexate; TAC, tacrolimus; GCs, glucocorticoids; SASP, salazosulfapyridine; Buc, bucillamine; IGU, iguratimod; AZA, azathioprine.

**Table 2:** Comparison of clinical characteristics and treatment between UIP-like pattern and non-UIP-like pattern groups.

Characteristic	UIP-like Pattern Group (*n* = 16)	Non-UIP-like Pattern Group (*n* = 22)	*p* Value
Age, years	74.5 (69–79.5)	72 (67–78.3)	0.564
Female sex, *n* (%)	11 (69)	13 (59)	0.735
Disease duration of RA, months	40.8 (6.3–156.6)	41.2 (10.5–115.5)	0.988
Smoking history, *n* (%)	9 (56)	9 (41)	0.512
RF positive, *n* (%)	16 (100)	16 (72.7)	0.030 *
RF, IU/mL	248 (78–555.3)	97 (52.5–642.5)	0.601
ACPA positivity, *n* (%)	16 (100)	17 (77.2)	0.061
ACPA, U/mL	393.5 (210.3–500)	290 (107.9–450)	0.165
RA disease activity indicator:			
DAS28-CRP	3.6 (2.9–4.3) ^a^	3.7 (3–4.2) ^b^	1
SDAI	18 (11.3–20.1) ^a^	14 (10.6–19.6) ^b^	0.585
CDAI	16.2 (9.2–20) ^a^	13.7 (9.4–18.7) ^b^	0.785
ILD indicators:			
KL-6, U/mL	450.5 (337.5–643)	381.5 (216.5–625.3) ^c^	0.3
FVC, %	90.7 (64.1–100.7) ^d^	104.1 (90.9–117.5) ^e^	0.108
Total GGO score	6.5 (4.2–8.2)	4.2 (3–7.8)	0.242
Total fibrosis score	5.2 (4.4–7.3)	2.3 (1.6–3.5)	0.0003 **
Treatment for RA:			
MTX, *n* (%)	3 (18.8)	7 (31.8)	0.469
MTX dose, mg/week	4 (4–4)	8 (6–9)	0.562
TAC, *n* (%)	10 (62.5)	12 (54.6)	0.744
GCs, *n* (%)	5 (31.2)	8 (36.4)	1
GC dose, mg/day	5 (4–8.8)	6.75 (5–10)	0.365
Others, *n* (%)	SASP, 6 (38); Buc, 0 (0); IGU, 1 (6.2); AZA, 1 (6.2)	SASP, 9 (41); Buc, 3 (14); IGU, 2 (9.1); AZA, 1 (4.6)	

Continuous variables are shown as the median (interquartile range). * *p* < 0.05, ** *p* < 0.001. ^a^, 15 patients; ^b^, 21 patients; ^c^, 16 patients; ^d^, 14 patients; ^e^, 9 patients. RA, rheumatoid arthritis; ILD, interstitial lung disease; RF, rheumatoid factor; ACPA, anti-cyclic citrullinated peptides antibody; CRP, C-reactive protein; DAS28-CRP, Disease Activity Score in 28 Joints using C-reactive Protein; SDAI, Simplified Disease Activity Index; CDAI, Clinical Disease Activity Index; KL-6, Krebs von den Lungen-6; MTX, methotrexate; TAC, tacrolimus; GCs, glucocorticoids; SASP, salazosulfapyridine; Buc, bucillamine; IGU, iguratimod; AZA, azathioprine.

**Table 3:** Results of logistic regression analyses of influencing factors of GGO reduction in chest HRCT findings.

	Univariable Analysis			Multivariable Analysis		
Risk Factors	Crude Odds Ratio	95% CI	*p*	Adjusted Odds Ratio	95% CI	*p*
GCs use (Ref: non-use)	1.524	0.32–7.15	0.595	2.883	0.33–24.85	0.324
TAC use (Ref: non-use)	1.458	0.32–6.46	0.619	1.890	0.36–9.80	0.441
MTX use (Ref: non-use)	1.275	0.24–6.70	0.775	1.712	0.26–11.2	0.574
RF positive (Ref: negative)	1	0.08–12.40	1	1.636	0.09–29.67	0.735
Smoking (Ref: non-smoking)	0.833	0.20–3.56	0.805	0.466	0.06–3.35	0.436

The odds ratios of GGO reduction in the chest HRCT findings were derived from univariable and multivariable logistic regression analyses. CI, confidence interval; Ref, reference; RA, rheumatoid arthritis; ILD, interstitial lung disease; GCs, glucocorticoids; TAC, tacrolimus; MTX, methotrexate; RF, rheumatoid factor; HRCT, high-resolution computed tomography.

## Data Availability

Regarding the submission of raw data, because it is difficult to protect personal information, the data will be disclosed only when reasonable requests are received. To request data from this study, the corresponding author should be contacted.

## Supplementary Materials

The following supporting information can be downloaded at: https://www.mdpi.com/article/10.3390/jcm13195871/s1, Figure S1. Changes in RA disease activities before and 1 year after the start of ABT administration. Paired analysis is performed using Wilcoxon’s signed-rank test. Statistical significance is set at *p* < 0.05. ABT, abatacept; Before, before ABT administration; After, 1 year after the start of ABT administration; DAS28-CRP, Disease Activity Score in 28 Joints using C-reactive Protein; SDAI, Simplified Disease Activity Index; CDAI, Clinical Disease Activity Index. Figure S2. Changes in chest HRCT scores before and 1 year after the start of ABT administration categorized by MTX use (A), non-use (B), smoking (C), and non-smoking (D). Paired analysis is performed using Wilcoxon’s signed-rank test. Statistical significance is set at *p* < 0.05. ABT, abatacept; MTX, methotrexate; Before, before ABT administration; After, 1 year after the start of ABT administration; GGO, ground-glass opacity.
