# Supplementary material for: The Therapeutic Efficacy of Abatacept for Rheumatoid Arthritis-Associated Interstitial Lung Disease: Insights from a 12-Month Trial Using Semi-Quantitative Chest High-Resolution Computed Tomography Imaging
Source: J Clin Med. 2024 Oct 1;13(19):5871. doi: 10.3390/jcm13195871 (PMC11477086; doi:10.3390/jcm13195871)
Supplement: Supplementary file 1 [file jcm-13-05871-s001.zip › jcm-3213655-Supplementary materials.pdf]

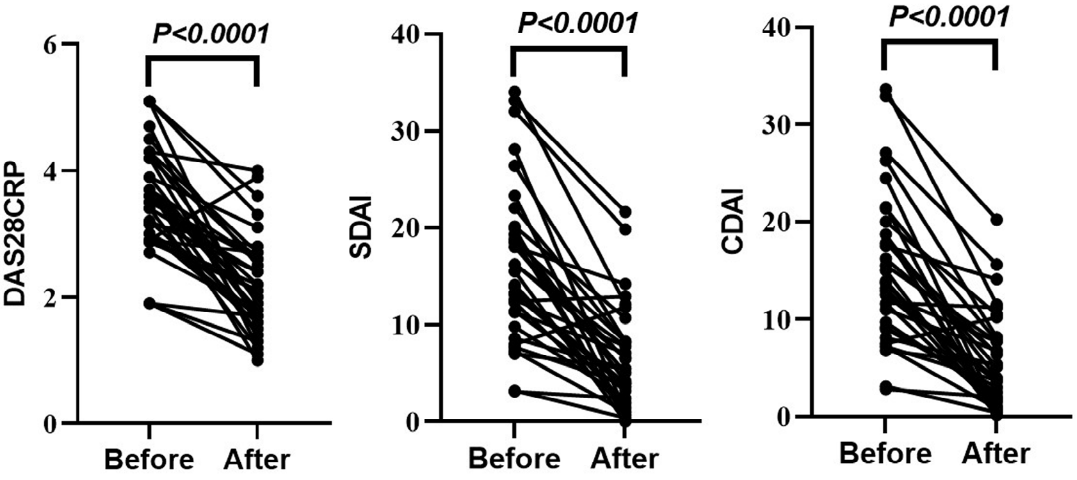

Figure S1. Changes in RA disease activities before and 1 year after the start of ABT administration. Paired analysis is performed using Wilcoxon's signed-rank test. Statistical significance is set at $p < 0.05$. ABT, abatacept; Before, before ABT administration; After, 1 year after the start of ABT administration; DAS28-CRP, Disease Activity Score in 28 Joints using C-reactive Protein; SDAI, Simplified Disease Activity Index; CDAI, Clinical Disease Activity Index.

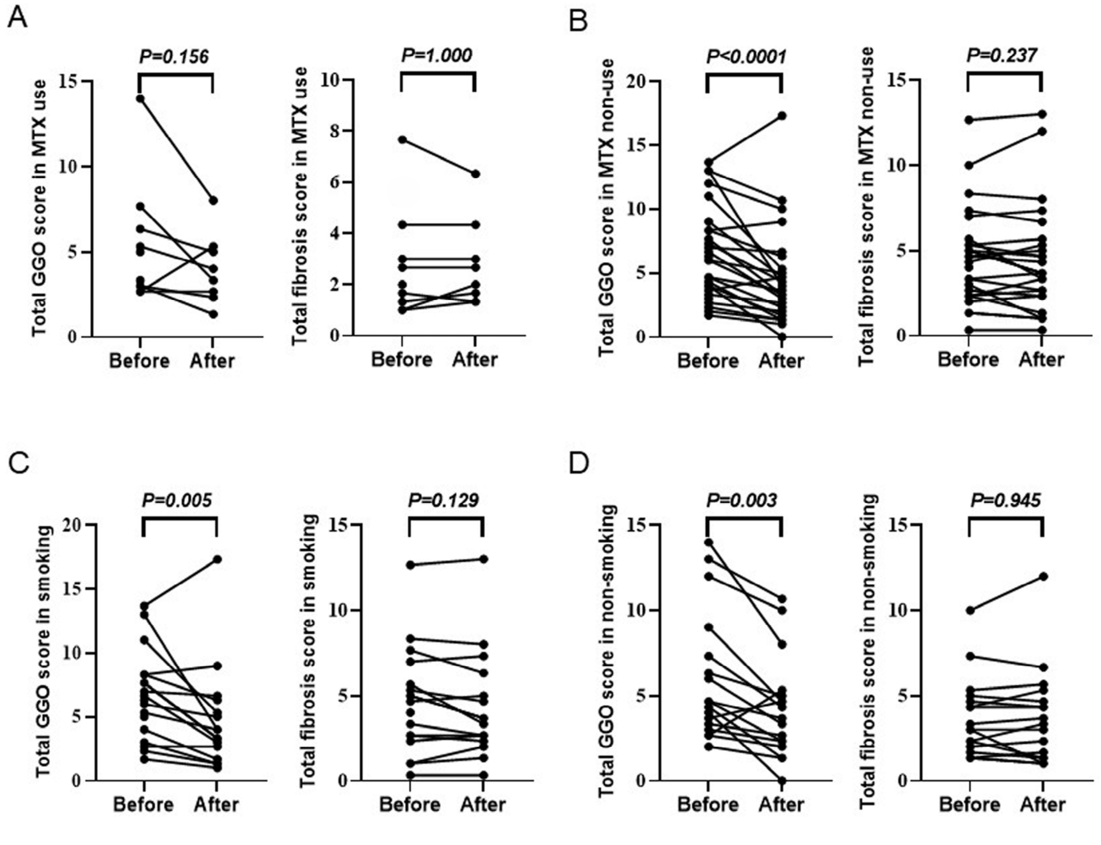

Figure S2. Changes in chest HRCT scores before and 1 year after the start of ABT administration categorized by MTX use (**A**), non-use (**B**), smoking (**C**), and non-smoking (**D**). Paired analysis is performed using Wilcoxon's signed-rank test. Statistical significance is set at $p < 0.05$. ABT, abatacept; MTX, metho-trexate; Before, before ABT administration; After, 1 year after the start of ABT administration; GGO, ground-glass opacity.
